# The beneficial effects of TAVI in mitral insufficiency

**DOI:** 10.1186/s12947-015-0040-5

**Published:** 2015-12-30

**Authors:** Marco Fabio Costantino, Ernesta Dores, Pasquale Innelli, Antonella Matera, Vincenza Santillo, Roberto Violini, Rosario Fiorilli, GianGiuseppe Cappabianca, Nicola Marraudino, Eugenio Picano, Giandomenico Tarsia

**Affiliations:** Cardiology Intensive Care Unit “Alta Specialità del Cuore” Department AORN San Carlo Hospital Potenza, Via Potito Petrone, 85100 Potenza, Italy; UO Emergency Care, San Giovanni di Dio Hospital Melfi –ASP, Potenza, Italy; UO Cardiology Intensive Care Unit, Villa d’Agri Hospital –ASP, Potenza, Italy; UO Interventional Cardiology Unit, S. Camillo Forlanini Hospital, Rome, Italy; UO Cardiac Surgery ,Ospedale di Circolo, Università dell’Insubria, Varese, Italy; UO Cardiac Surgery, Università di BARI, Bari, Italy; Institute of clinical physiology, CNR, Pisa, Italy

**Keywords:** Mitral regurgitation, Aortic stenosis, Transcatheter Aortic Valve Implantation (TAVI)

## Abstract

**Background:**

Previous studies have suggested that concomitant mitral regurgitation (MR) is a risk factor for acute transcatheter aortic valve implantation (TAVI) failure, but may improve afterwards. Aim of this study was to assess the prevalence, clinical meaning and modifications of MR in patients undergoing TAVI.

**Methods:**

In a retrospective, two-center (Potenza-San Carlo and Roma- San Camillo) study, from January 2010 to June 2014 we enrolled 165 consecutive patients (age =80 ± 5 years, 74 males, Ejection Fraction 51 ± 9 %) referred for TAVI with either Medtronic Core-ReValving System (in 114 patients, 69 %) or balloon-expandable Edwards SAPIEN/SAPIEN XT (in 51 patients, 31 %). All patients underwent TTE and TEE assessment of MR (from 1, mild to 4 = severe according to ESC latest guidelines) with core lab reading by a single observer blinded to patient identity and status. Assessment was performed at baseline (24 h prior to intervention) and at 1, 6, 12 and 24 months.

**Results:**

Mild-to-Moderate MR (grade 1–2) was present in 137 patients and Moderate-to-Severe MR (grade 3–4) was present in 28 patients. No significant differences were seen comparing perioperative mortality and morbidity between the two groups. In the group of preoperative MR grade 3–4 the mean decrease from MR pre-TAVI to MR at 1 month post-TAVI was 0.464 (*p* < 0.0001) and this improvement was persistent at 6 months (*p* < 0.0001) and at 12 months (*p* < 0.0001), with partial benefit loss at 1 and 2 years. The mean difference from Left Atrial volume post-TAVI at 1 month was 16.5 ml (*p* < 0.0001) and this improvement was persistent at 12 months 12.12 ml (*p* < 0.0001).

**Conclusions:**

TAVI effectively treats the aortic valve but as a beneficial by product also ameliorates concomitant MR. The presence of moderate-to-severe MR does not increase the acute risk of failure of TAVI. In successful procedures, the MR improves immediately and persistently.

## Background

Mitral regurgitation (MR) is a common finding in patients with aortic stenosis (AS). At the time of aortic valve replacement (AVR) up to two thirds of patients with AS have varying degrees of MR [1]. Most published studies on valvular heart disease have focused on either regurgitant or stenotic single valve disease. Data on multi-valve disease are scarce. As result, North American and European guidelines offer limited insight with respect to management of multivalve disease. Those recommendations that are made are largely based on small studies or on expert consensus opinion (Grade C).

A decrease in MR severity is common following isolated AVR [2–4]. Early improvement might result from acute reverse left ventricular (LV), including a reduction in LV end-diastolic volume and a decrease in mitral tethering forces [5, 6].

Transcatheter valve therapies are a feasible alternative to conventional open-heart surgery in many patients with valvular disease. For AS, transcatheter aortic valve implantation (TAVI) is the standard of care in inoperable patients and an alternative to SVAR in high-surgical risk patients [7–13]. However , TAVI is still a relatively novel technology, and short and long term morbidity and mortality after TAVI remain significant [14]. There is substantial interest in the identification and modification of factors influencing morbidity and mortality after TAVI.

Recently, Chakravarty et al. [14], have reported that moderate-severe MR is present in 20 % to 30 % of patients who underwent TAVI and constitutes a significant coexisting valvular heart disease burden [15–21]. In this meta-analysis, the severity of MR improved after TAVI in 61 ± 6.0 % of patients, but baseline moderate-severe MR and significant residual MR after TAVI are associated with an increase in mortality after TAVI and represent an important group to target with medical or transcatheter therapies in the future [14]. Therefore the aim of this retrospective, observational, two-center study was to evaluate the improvement of mitral regurgitation in patients undergoing TAVI with concomitant MR.

## Methods

### Study design and patient population

From January 2010 to June 2014, 165 consecutive patients affected by severe aortic stenosis underwent TAVI either using Medtronic Core-ReValving System (in 114 patients, 69 %) or using balloon-expandable Edwards SAPIEN/SAPIEN XT (in 51 patients, 31 %) at Potenza-San Carlo Hospital and Roma-San Camillo Hospital.

All patients were evaluated for TAVI by the local heart team, which included a clinical cardiologist, an interventional cardiologist, a cardiac surgeon, and a cardiac anesthesiologist. The evaluation of the heart team led to the indication for TAVI after careful assessment of all the clinical/anatomic conditions determining a higher risk of mortality/morbidity after surgery. In all patients scheduled for TAVI who gave written consent for the procedure follow-up was scheduled at 1, 6, 12 and 24 months. Patients were followed up by means of outpatient clinics and regular contact with clinical cardiologist. At any follow-up time all patients are underwent a clinical exam and echocardiographic study.

To define the events in the follow-up we referred to the current standard for definition of the events in TAVI represented by VARC-2 criteria [22].

### Device and procedure

Arterial access (femoral,radial), percutaneous puncture, or surgical exposure was also determined

on the basis of the panel of preoperative imaging tests, which included in all cases both angiography and computed tomography scan. After the procedure, all patients were managed in an intensive care unit or coronary care unit for at least one day.

### Data collection and definitions

Transthoracic and TEE echocardiography was performed before TAVI, after TAVI, and at 1, 6, 12 and 24 months by a senior cardiologist. MR severity was graded as no/mild (0/1), moderate (2), moderate-severe(3) or severe (4), [23]*.* Mitral annular calcification, prolapse and thickening was reported according to the guidelines [23]. MR type has been classified as organic (primary) or functional (secondary). Organic MR is attributable to intrinsic valvular disease, whereas functional MR is caused by regional or global left ventricle (LV) remodeling without structural abnormalities of the valve apparatus. A medical record reporting a fatality was available in 13 patients (7.9 %); echocardiographic follow-up was available in 152 patients. Basal characteristic of overall population are shown in Table 1.

**Table 1:** Basal characteristic of overall population according to MR grade groups

	All	Preoperative MR	Preoperative MR	*p* value
		Grade 3–4	Grade 1–2	
Patients	165	28	137	
Demographics				
Males	74 (44.8 %)	19 (67.9 %)	55 (40.1 %)	0.01
Mean Age (years)	80.2 ± 5.6	81 ± 5.2	79.9 ± 5.6	0.38
Weight (kg)	67.1 ± 10.8	71.6 ± 7.2	66.2 ± 11.1	0.01
Height (m)	1.62 ± 0.07	1.67 ± 0.05	1.61 ± 0.08	0.0005
Body surface area (m^2^)	1.7 ± 0.2	1.81 ± 0.08	1.69 ± 0.16	0.0005
Ejection Fraction (%)	51 ± 9.3	39.8 ± 7.5	53.2 ± 7.9	<0.0001
Aortic valve				
Peak gradient (mmHg)	75.5 ± 17.5	63.8 ± 20.8	77.9 ± 15.7	<0.0001
Mean gradient (mmHg)	44.8 ± 8.2	39.5 ± 10.5	45.8 ± 7.2	0.0002
Aortic valve area (cm^2^)	0.59 ± 0.9	0.58 ± 0.08	0.6 ± 0.1	0.34
Mitral valve				
MR mechanism				
Degenerative	118 (71.5 %)	7 (25 %)	111 (81 %)	<0.0001
Functional	47 (28.5 %)	21 (75 %)	26 (19 %)	<0.0001
Leaflets disease				
Calcifications	55 (33.3 %)	2 (7.1 %)	53 (38.7 %)	0.0008
Thickening	63 (38.2 %)	5 (17.9 %)	58 (42.3 %)	0.02
Prolapse	7 (4.2 %)	2 (7.1 %)	5 (3.6 %)	0.33
Mitral regurgitation [1–4]	1.9 ± 0.7	3.1 ± 0.3	1.6 ± 0.5	<0.0001
MR grade I	54 (32.7 %)	54 (192.9 %)	0 (0 %)	n.c
MR grade II	83 (50.3 %)	83 (296.4 %)	0 (0 %)	n.c.
MR grade III	25 (15.2 %)	0 (0 %)	25 (18.2 %)	n.c.
MR grade IV	3 (1.8 %)	0 (0 %)	3 (2.2 %)	n.c.
EROA (mm2)	24.9 ± 8	38.5 ± 6.7	22.1 ± 4.7	<0.0001
Vena contracta (mm)	36.1 ± 11.8	55.4 ± 9.4	32.1 ± .7.5	<0.0001
Regurgitant volume (ml)	35.1 ± 10.2	50.8 ± 7	31.9 ± 7.3	<0.0001
Regurgitant fraction (%)	33.6 ± 9.9	48.6 ± 6.4	30.4 ± 7.2	<0.0001
LA volume (ml)	64.2 ± 14.3	79.6 ± 17.7	61 ± 11.1	<0.0001
Procedure				
Prosthesis model				
Corevalve	114 (69.1 %)	19 (67.9 %)	95 (69.3 %)	0.99
Sapien	51 (30.9 %)	9 (32.1 %)	42 (30.7 %)	0.99
Valve size (mm)	26.8 ± 2.5	27.8 ± 2.3	26.6 ± 2.4	0.02

### Statistical analysis

Categorical variables were presented as absolute numbers and percentages and compared using chi-square test. Continuous variables were presented as mean ± standard deviation and were compared using t-test.

For echocardiographic data, a two way analysis for repeated measures (between the two groups and among different times) was performed using the Linear Mixed Model; individual comparison between groups for each parameter at different times was carried using unpaired t-test with post-hoc Bonferroni correction.

Kaplan and Meier curves were used to calculate the survival probability. Cox proportional hazard model was instead used to perform univariate analysis of mortality. Since only a single factor (female sex) was found to have a p value ≤ 0.10, multivariate analysis could not be carried.

All P values reported are 2 sided, and a value of *P* < 0.05 was considered significant. All data were processed with the Statistical Package for Social Sciences, version 21 (SPSS, Chicago, IL).

## Results

Moderate-Severe MR (Grade 3–4) at the time of the procedure was present in 28 pts (17 %).

Mild-Moderate MR (Grade 1–2) was present in 137 pts (83 %).

Patients with concomitant grade 3–4 MR appear quite different from patients with grade 1–2 MR.

Patients with Moderate-Severe MR (Grade 3–4) had a lower LV function compared with patients with Mild-Moderate MR (Grade 1–2) (39.8 ± 7.5 % versus 53.2 ± 7.9 %; *p* < 0.0001), a lower trans-aortic Mean gradient (39.5 ± 10.5 mmHg versus 45.8 ± 7.2 mmHg; *p* = 0.002) and a lower trans-aortic Peak gradient (63.8 ± 20.8 mmHg versus 77.9 ± 15.7 mmHg; *p* < 0.0001).

In patients with grade 3–4 MR, the aetiology of mitral valve disease is predominantly functional (75 %) as also confirmed by the significantly lower incidence of structural changes of the mitral leaflets (Calcifications 7.1 % and Thickening 17.9 %).

Although preoperative characteristics are different, there aren’t statistically significant differences, as shown in Table 2, regarding mortality at 30 days between two groups (3 patients, 2.1 % for MR grade 1–2 group and 0 patients, 0 % for MR grade 3–4 group; *p* = 0.99) and incidence of peri-procedure complications: bleeding (4 patients, 2.9 % for MR grade 1–2 group and 2 patients, 7.1 % for MR grade 3–4 group; *p* = 0.26); neurological complications (7 patients, 5.1 % for MR grade 1–2 group and 0 patients, 0 % for MR grade 3–4 group; *p* = 0.60).

**Table 2:** Post operative results, according to MR grade groups

	All	Preoperative MR	Preoperative MR	*p* value
		Grade 3–4	Grade 1–2	
Patients	165	28	137	
30 days mortality	3 (1.8 %)	0	3 (2.1 %)	0.99
Bleeding	6 (3.6 %)	2 (7.1 %)	4 (2.9 %)	0.26
Neurological complications	7 (4.2 %)	0	7 (5.1 %)	0.60

### Analysis of echocardiographic parameters

**Fig. 1:**
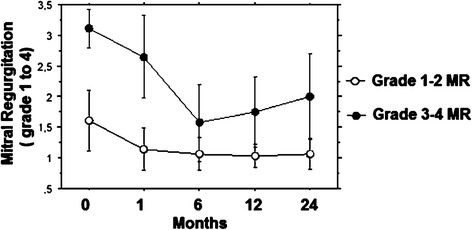
Different value of degree of mitral regurgitation at 0, 6, 12 and 24 months of follow-up in the two groups

In the overall population, MR score went from 2.1 ± 0.6 to 1.6 ± 0.8 (*p* < 0.001) at the end of follow-up. Figure 1 depicts the comparison between the two study groups regarding the degree of MR before and following TAVI up to two years: two way repeated analysis showed a significant difference on the degree of MR between the groups (F = 573.1; *p* < 0.001) and across the different times (F = 72.3; *p* < 0.0001). For the group with preoperative MR grade 1–2 no significant difference was noted comparing the degree of preoperative MR with any of the postoperative timeframes.

In the group with preoperative MR grade 3–4 the mean decrease from MR pre-TAVI to MR at 1 month post-TAVI was 0.464 (*p* < 0.0001) and this improvement was persistent at 6 months (*p* < 0.0001), 12 months (*p* < 0.0001) and 24 months (*p* < 0.0001), with partial benefit loss at 1 and 2 years (Fig. 1).

**Fig. 2:**
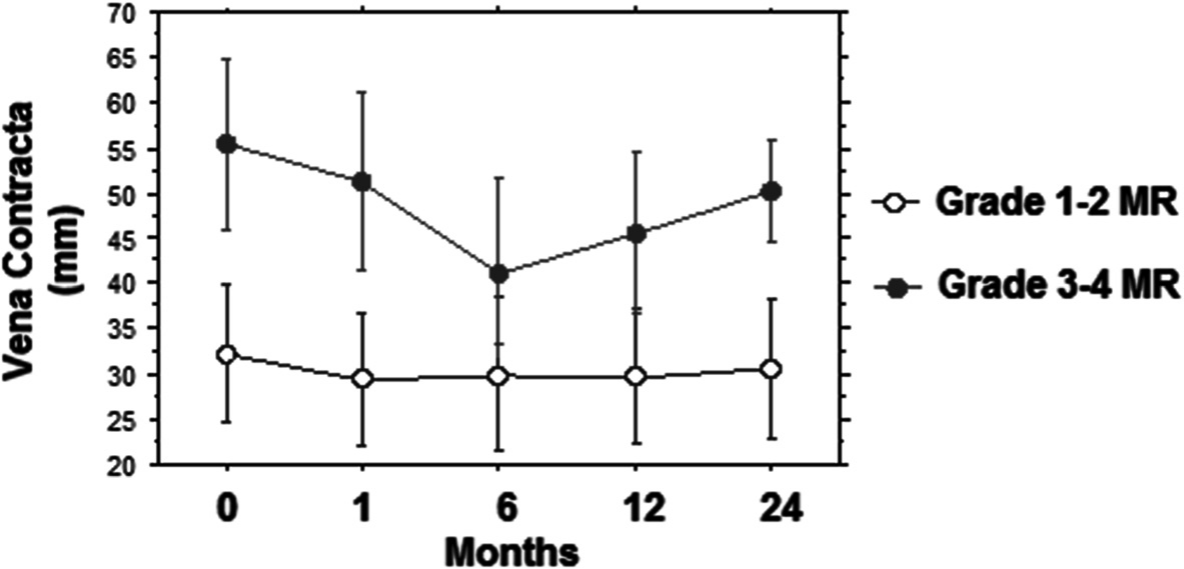
Different value of MR Vena Contracta (VC) at 0, 6, 12 and 24 months of follow-up in the two groups

Figure 2 depicts the comparison between the two study groups regarding the degree of Vena Contracta (VC) before and following TAVI up to two years: two way repeated analysis showed a significant difference on the degree of VC between the groups (F = 431.0; *p* < 0.001) and across the different times (F = 6.4; *p* = 0.0005). For the group with preoperative MR grade 1–2 no significant difference was noted comparing the degree of preoperative VC with any of the postoperative timeframes.

In the group with preoperative MR grade 3–4 the mean decrease from VC pre-TAVI to VC at 1 month post-TAVI was 4.1 mm (*p* < 0.0001) and this improvement was persistent at 6 months , 14.4 mm (*p* < 0.0001), 12 months (*p* < 0.0001) and 24 months (*p* < 0.0001), (Fig. 2).

**Fig. 3:**
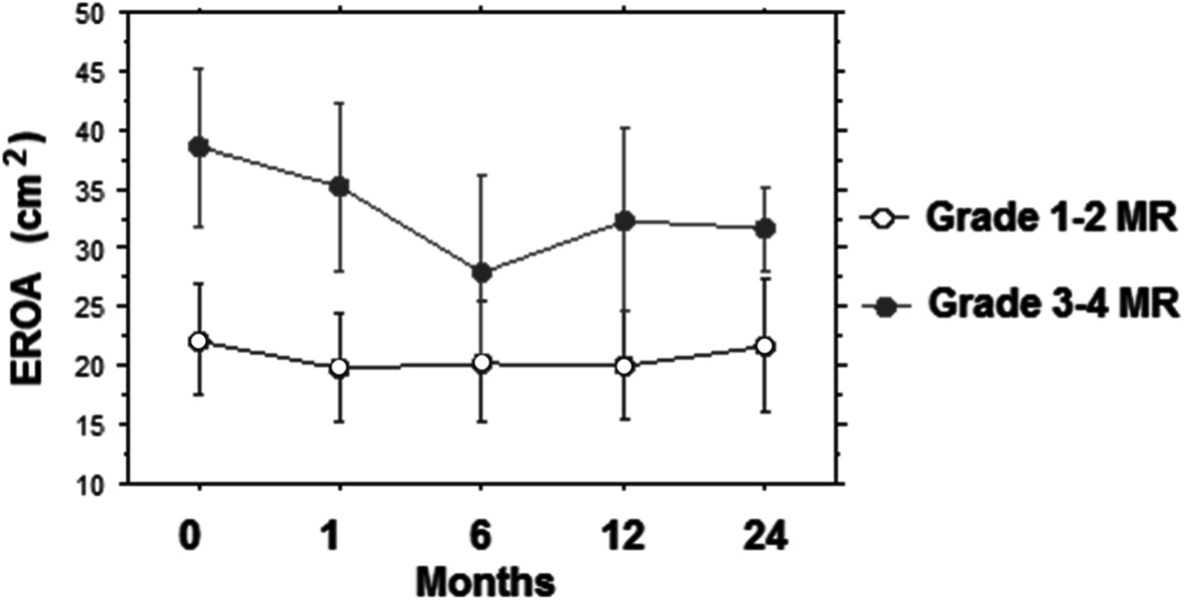
Different value of MR EROA at 0, 6, 12 and 24 months of follow-up in the two groups

Figure 3 depicts the comparison between the two study groups regarding the degree of EROA before and following TAVI up to two years: two way repeated analysis showed a significant difference on the degree of EROA between the groups (F = 477.5; *p* < 0.001) and across the different times (F = 8.9; *p* < 0.0001). For the group with preoperative MR grade 1–2 no significant difference was noted comparing the degree of preoperative EROA with any of the postoperative timeframes.

In the group with preoperative MR grade 3–4 the mean decrease from EROA pre-TAVI to EROA at 1 month post-TAVI was 3.42 mm^2^ (*p* < 0.0001) and this improvement was persistent at 6 months (*p* < 0.0001), 12 months (*p* < 0.0001) and 24 months (*p* < 0.0001), (Fig. 3).

**Fig. 4:**
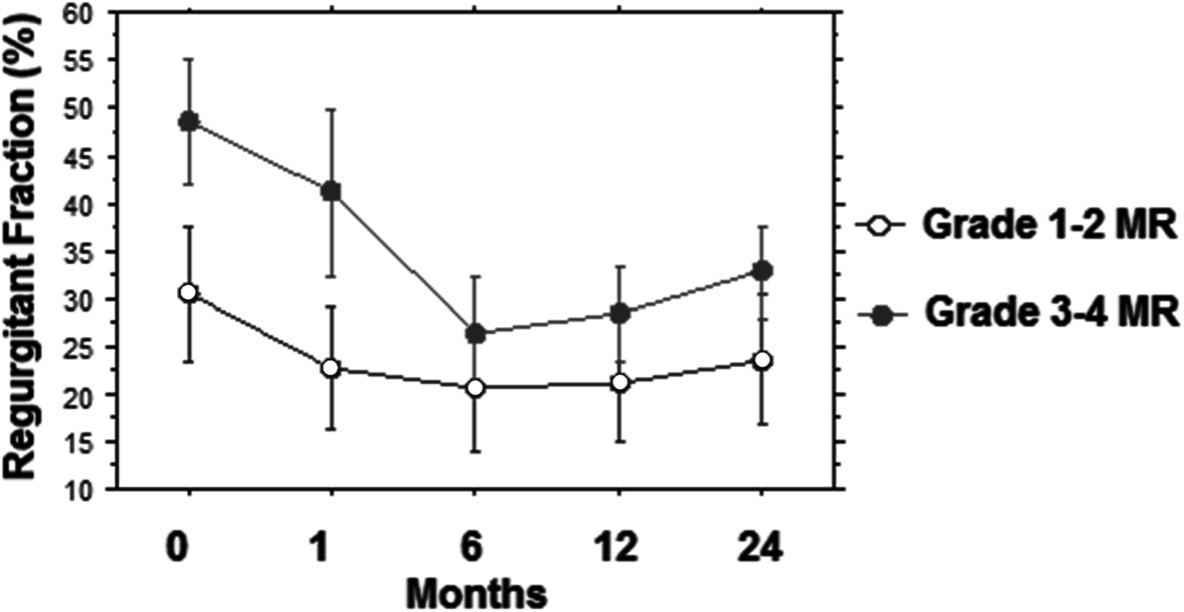
Different value of MR Regurgitant Fraction (RF) at 0, 6, 12 and 24 months of follow-up in the two groups

Figure 4 depicts the comparison between the two study groups regarding the degree of Regurgitant fraction (RF) before and following TAVI up to two years: two way repeated analysis showed a significant difference on the degree of RF between the groups (F = 285.2; *p* < 0.001) and across the different times (F = 68.3; *p* < 0.0001). For the group with preoperative MR grade 1–2 no significant difference was noted comparing the degree of preoperative RF with any of the postoperative timeframes.

In the group with preoperative MR grade 3–4 the mean decrease from RF pre-TAVI to RF at 1 month post-TAVI was 7.5 % (*p* < 0.0001) and this improvement was persistent at 6 months (*p* < 0.0001), 12 months (*p* < 0.0001) and 24 months (*p* < 0.0001), (Fig. 4).

**Fig. 5:**
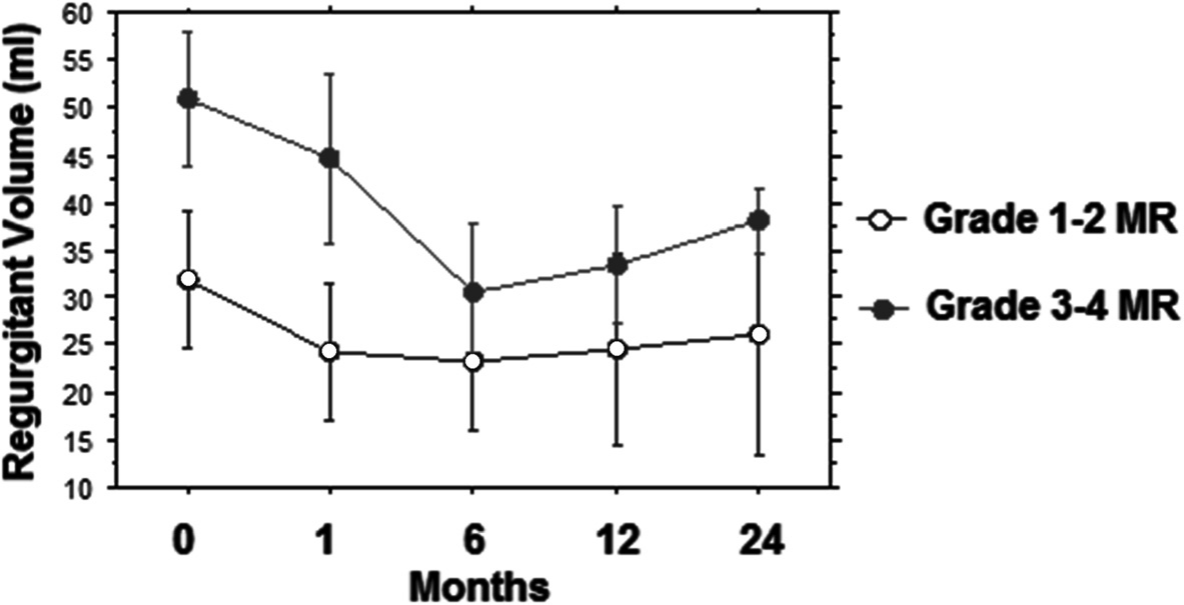
Different value of MR Regurgitant Volume (RV) at 0, 6, 12 and 24 months of follow-up in the two groups

Figure 5 depicts the comparison between the two study groups regarding the degree of Regurgitant volume (RV) before and following TAVI up to two years: two way repeated analysis showed a significant difference on the degree of RV between the groups (F = 284.4; *p* < 0.001) and across the different times (F = 35.9; *p* < 0.0001). For the group with preoperative MR grade 1–2 no significant difference was noted comparing the degree of preoperative RV with any of the postoperative timeframes.

In the group with preoperative MR grade 3–4 the mean decrease from RV pre-TAVI to RV at 1 month post-TAVI was 6.2 (*p* < 0.0001) and this improvement was persistent at 6 months (*p* < 0.0001), 12 months (*p* < 0.0001) and 24 months (*p* < 0.0001), (Fig. 5).

**Fig. 6:**
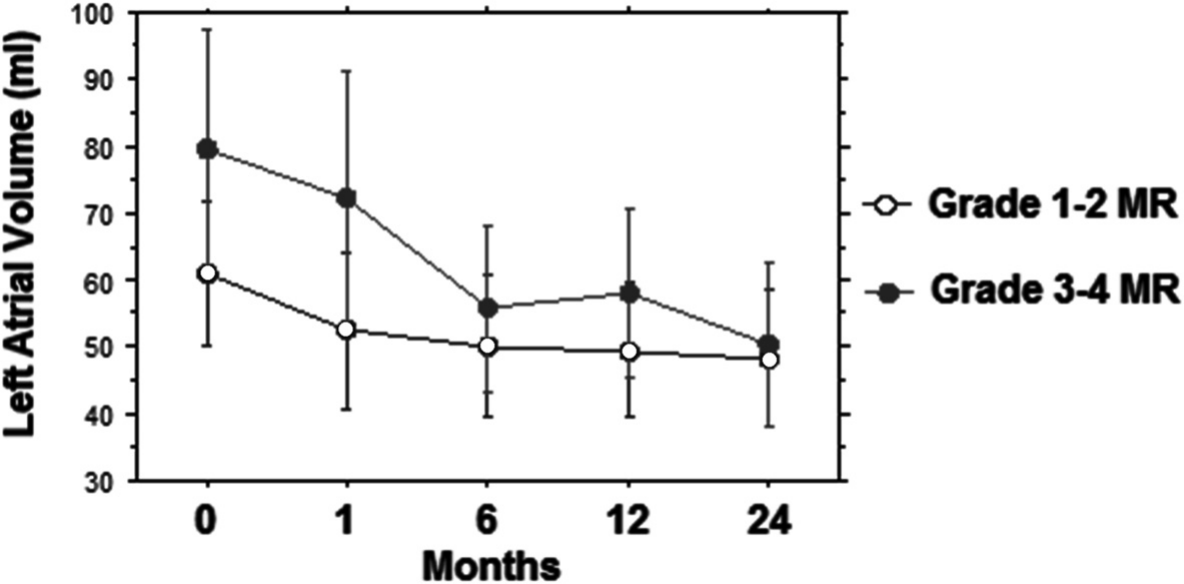
Different value of Left Atrial Volume (ml) at 0, 6, 12 and 24 months of follow-up in the two groups

Figure 6 depicts the comparison between the two study groups regarding the Left atrial volume (LA) of MR before and following TAVI up to two years: two way repeated analysis showed a significant difference on the degree of LA between the groups (F = 103.4; *p* < 0.001) and across the different times (F = 33.7; *p* < 0.0001). For the group with preoperative MR grade 1–2 no significant difference was noted comparing the degree of preoperative LA with any of the postoperative timeframes.

In the group with preoperative LA grade 3–4 the mean decrease from LA pre-TAVI to MR at 1 month post-TAVI was 16.5 (*p* < 0.0001) and this improvement was persistent at 6 months (*p* < 0.0001), 12 months (*p* < 0.0001) and 24 months (*p* < 0.0001), (Fig. 6).

### Follow-up survival

The mean follow-up was 1.1 ± 0.6 years and all patients (100 %) completed at the follow-up.

**Fig. 7:**
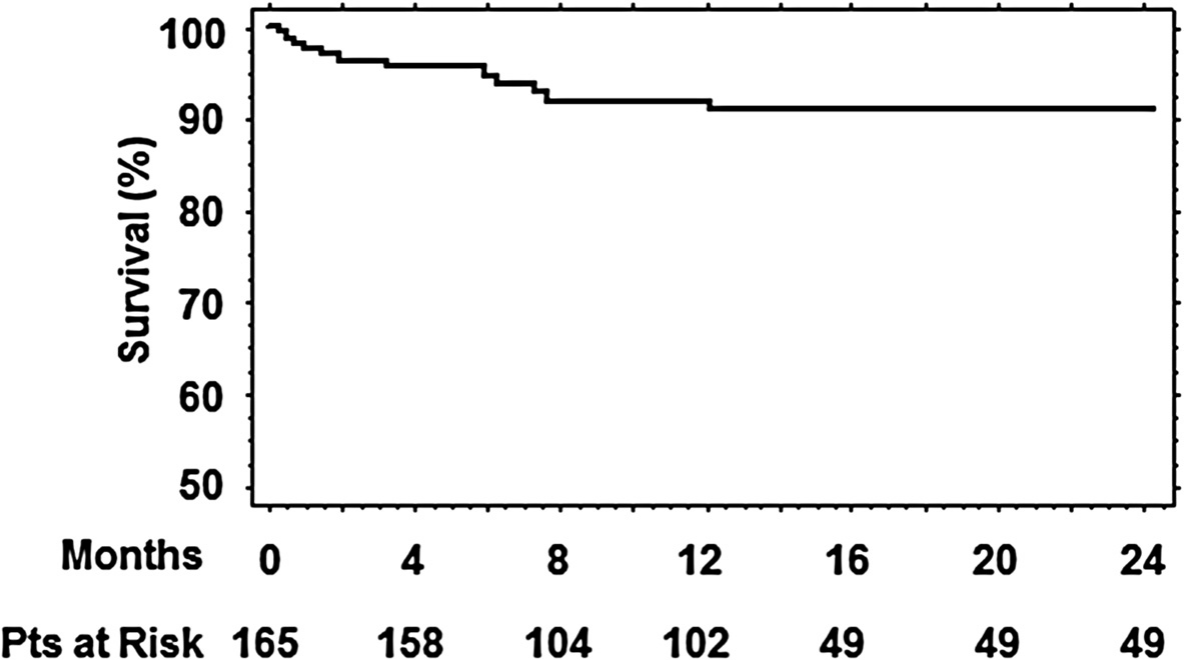
2 years survival curve for both group

Figure 7 shows the 2 years survival curve for both groups: 6 months of 94.5 ± 1.8 %, at 12 months of 90.9 ± 2.5 % and 24 months of 90.0 ± 2.5 %. The follow up mortality (10 patients, 6.0 %) occurred exclusively in the grade 1–2 MR group. Cox hazard model showed that the only predictive factor of follow-up mortality was female sex (HR:11.5, 95 % CI:1.4-85.2, *p* = 0.02).

## Discussion

Compared with patients with mild baseline MR, those with moderate or severe MR have a worse baseline clinical characteristics (Low EF, Dilated LV), but post-procedural mortality and morbidity are similar in two groups. In fact, in patients with moderate or severe baseline MR, the MR severity improves in post procedural follow-up by overall echo-indices (vena contracta, Regurgitant volume, Regurgitant fraction) : in 24 months follow-up the post procedural values are lower than pre-procedural status.

The presence of 2 different devices might add complexity to the interpretation of the scenario. It has been postulated that differences in the structure of the 2 devices (Core Valve and Sapien) may imply a different risk of mitral valve dysfunction; in other words, the longer nitinol frame of the Core Valve could mechanically interfere with the anterior leaflet of the mitral apparatus, especially in the presence of a low implantation [24]. Our data actually rule out this phenomenon because the incidence of worsened MR was quite low and no difference was observed in the low implantation rate between those with and those without a worsened MR. Similarly there are no differences, in our population, between organic and functional MR.

About follow-up survival, our data showed that female sex was the only predictive factor of mortality. This is not surprising since 12 out 13 deaths were females.

Few and contrasting results have been reported in the literature in terms of the prognostic significance and magnitude of MR changes following TAVI. A sub-analysis of the PARTNER trial [25] reported that preoperative moderate or severe MR (mostly moderate) was associated with increased two-year mortality after surgical AVR, but not after TAVI, suggesting that TAVI may be a reasonable option in selected high-risk patients with combined aortic and mitral valve disease. As with the PARTNER sub-analysis, D’Onofrio et al. [26] found that moderate or severe MR did not appear to be a significant risk factor for in-hospital mortality after TAVI. In contrast, Toggweiler et al. [27] found that moderate or severe MR in patients undergoing TAVI was associated with a higher early, but not late, mortality rate.

The results of our study are consistent with the previous literature. Recently a meta-analysis of 8 studies involving 8927 patients [14] evaluating the impact of MR on outcomes after TAVI found that (a) significant MR at baseline is associated with increased mortality after TAVI; (b) the cause of MR (functional or degenerative) or the type of transcatheter heart valve (Edwards valve or CoreValve) does not affect mor- tality after TAVI; (c) MR severity improves in up to 2/3 of patients after TAVI; and (d) moderate-severe residual MR is associated with increased mortality after TAVI.

The mechanism of improvement in MR severity is clearly multifactorial [17]. The improved aortic valve performance, with the subsequent reduction of the afterload, is conceivably expected to reduce the pathological retrograde flow through the mitral valve. It can also be presumed that the resolution of aortic stenosis may facilitate the achievement of a better hemodynamic balance by reducing the neurohormonal activation caused by the heart failure status. The treatment of the aortic stenosis may also contribute to the restoration of the proper geometry of the LV contraction, which may in turn contribute to improved function of the mitral valve apparatus, in particular when the concomitant MR is a functional type.

## Conclusions

TAVI effectively treats the aortic valve but as a beneficial by product also ameliorates concomitant MR. The presence of moderate-to-severe MR does not increase the acute risk of failure of TAVI. In successful procedures, the MR improves immediately and persistently.
